# Differential diagnosis of pancreatic cystic neoplasms through a radiomics-assisted system

**DOI:** 10.3389/fonc.2022.941744

**Published:** 2022-12-16

**Authors:** Zhenglin Dong, Xiahan Chen, Zhaorui Cheng, Yuanbo Luo, Min He, Tao Chen, Zijie Zhang, Xiaohua Qian, Wei Chen

**Affiliations:** ^1^ Department of Biliary-Pancreatic Surgery, Renji Hospital, School of Medicine, Shanghai Jiao Tong University, Shanghai, China; ^2^ Department of orthopedics, Shanghai Ninth People’s Hospital, Shanghai Jiao Tong University School of Medicine, Shanghai, China; ^3^ School of Biomedical Engineering, Shanghai Jiao Tong University, Shanghai, China; ^4^ Department of Rheumatology, Renji Hospital, School of Medicine, Shanghai Jiao Tong University, Shanghai, China; ^5^ Department of Otorhinolaryngology, Shanghai Ninth People’s Hospital, Shanghai Jiao Tong University School of Medicine, Shanghai, China; ^6^ Department of Liver Surgery, Renji Hospital, School of Medicine, Shanghai Jiao Tong University, Shanghai, China

**Keywords:** radiomics, computed tomography, pancreatic cystic neoplasm, differential diagnosis, ternary classification model

## Abstract

Pancreatic cystic neoplasms (PCNs) are a group of heterogeneous diseases with distinct prognosis. Existing differential diagnosis methods require invasive biopsy or prolonged monitoring. We sought to develop an inexpensive, non-invasive differential diagnosis system for PCNs based on radiomics features and clinical characteristics for a higher total PCN screening rate. We retrospectively analyzed computed tomography images and clinical data from 129 patients with PCN, including 47 patients with intraductal papillary mucinous neoplasms (IPMNs), 49 patients with serous cystadenomas (SCNs), and 33 patients with mucinous cystic neoplasms (MCNs). Six clinical characteristics and 944 radiomics features were tested, and nine features were finally selected for model construction using DXScore algorithm. A five-fold cross-validation algorithm and a test group were applied to verify the results. In the five-fold cross-validation section, the AUC value of our model was 0.8687, and the total accuracy rate was 74.23%, wherein the accuracy rates of IPMNs, SCNs, and MCNs were 74.26%, 78.37%, and 68.00%, respectively. In the test group, the AUC value was 0.8462 and the total accuracy rate was 73.61%. In conclusion, our research constructed an end-to-end powerful PCN differential diagnosis system based on radiomics method, which could assist decision-making in clinical practice.

## Introduction

Pancreatic cystic lesion (PCL) is one of the most common pancreatic diseases with a prevalence rate as high as 42% (1), and common pancreatic cystic neoplasms (PCN) accounts for 90.5% PCL (2). PCN is identified as a group of heterogeneous diseases with diverse characteristics and different prognosis (3). Serous cystadenoma (SCN), mucinous cystic neoplasm (MCN), and intraductal papillary mucinous neoplasm (IPMN) are the three main types of PCN, accounting for more than 85% of PCN (4). Because of the different rates of malignant transformation, the treatment principles of these three PCN subtypes recommended by the guidelines are variable (3).

Although modern modalities have been ubiquitously applied, the current diagnostic methods that can identify and evaluate PCN are still limited. In recent years, endoscopic ultrasonography–guided fine-needle aspiration (EUS-FNA) has been performed for cyst fluid analysis (5, 6). It is considered as a fairly sensitive tool for distinguishing PCN. However, EUS-FNA is an invasive method. Patients would suffer more pain than the non-invasive methods, such as computed tomography (CT) and MRI (7, 8). More importantly, CT and MRI have been widely applied as parts of health checkup. Compared with MRI, CT is even more widely used, is typically less expensive, and has less time for appointment. Unfortunately, SCNs, MCNs, and branch-duct IPMNs (BD-IPMNs) all demonstrate isolated cystic masses with low density cyst fluid and mild enhancement of the cyst wall on CT imaging, leading to a poor differential diagnosis and assessment even for an experienced radiologist (9). Moreover, the diagnostic accuracy rate of CT for PCN is between 40% and 81% (10–13). Thus, a higher diagnostic rate of contrast CT for PCN would extensively improve the total PCN screening rate.

Therefore, there is an urgent demand to develop a new non-invasive biomarker with a high accuracy in PCN diagnosis. Radiomics, sometimes referred to as “quantitative imaging”, is an emerging field focusing on disease auxiliary diagnosis or prognosis prediction and always integrates information such as genomic, transcriptome, and clinical data (14). After automatic feature extraction from images based on different algorithms, radiomics model is validated by support vector machine (SVM) or other classifiers. Through high-dimensional quantifiable features from images, radiomics can effectively and quantitatively evaluate the heterogeneity of images. Radiomics-assisted CT scan–based systems have ameliorated the accuracy of differential diagnosis in several organs, such as lung, central nervous system, rectum, liver, and pancreas (15–19). However, until now, relatively few studies have focused on PCN by analyzing SCNs, MCNs, and IPMNs simultaneously.

Thus, the aim of our study is to develop a non-invasive differential diagnosis system for PCNs based on imaging features in conjunction with patient’s clinical information.

## Materials and methods

### Workflow

**Figure 1:**
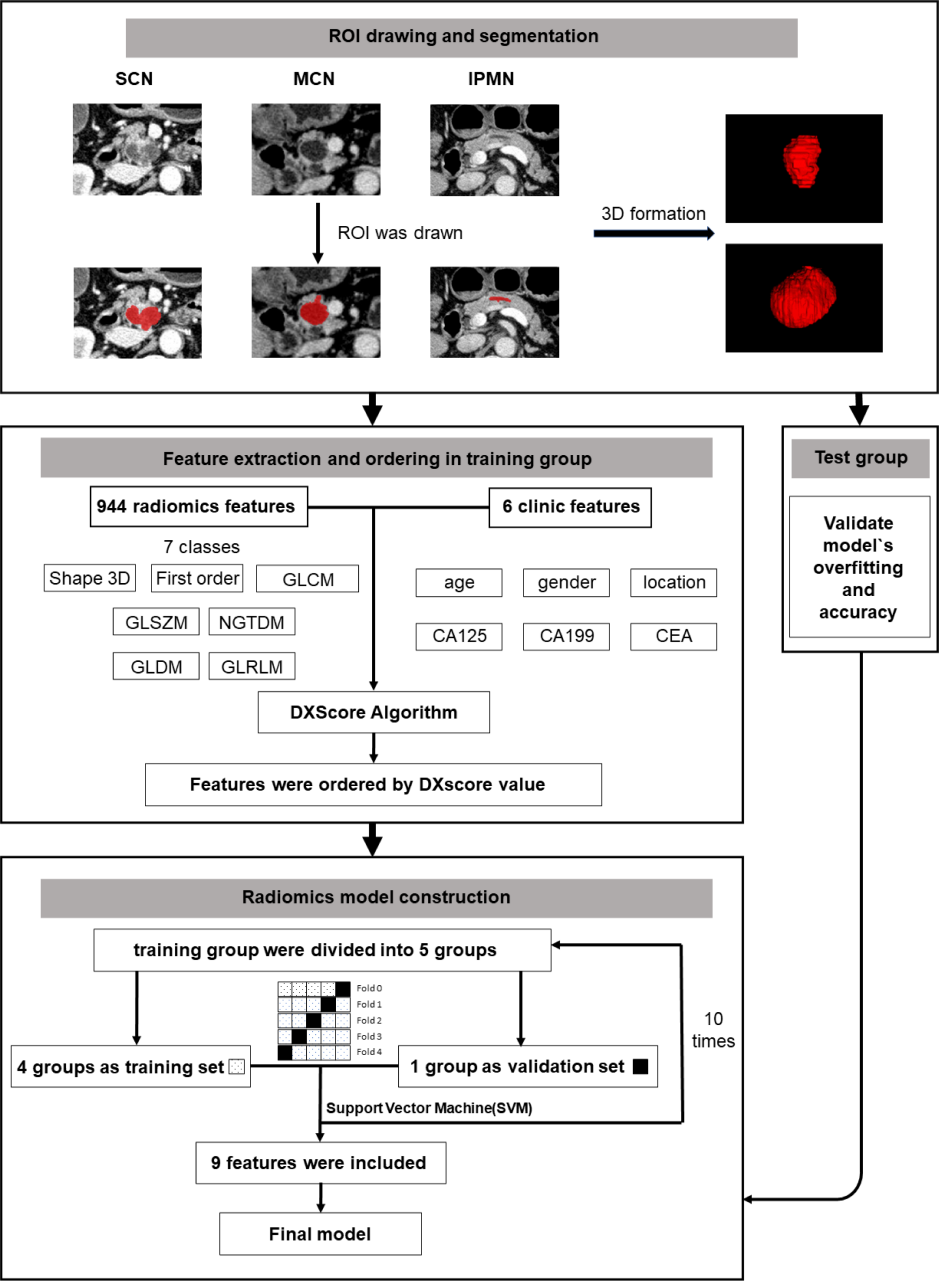
A general workflow of image processing and model development.

The brief workflow of image processing and model development was illustrated in **Figure 1**.

### Patient selection

**Figure 2:**
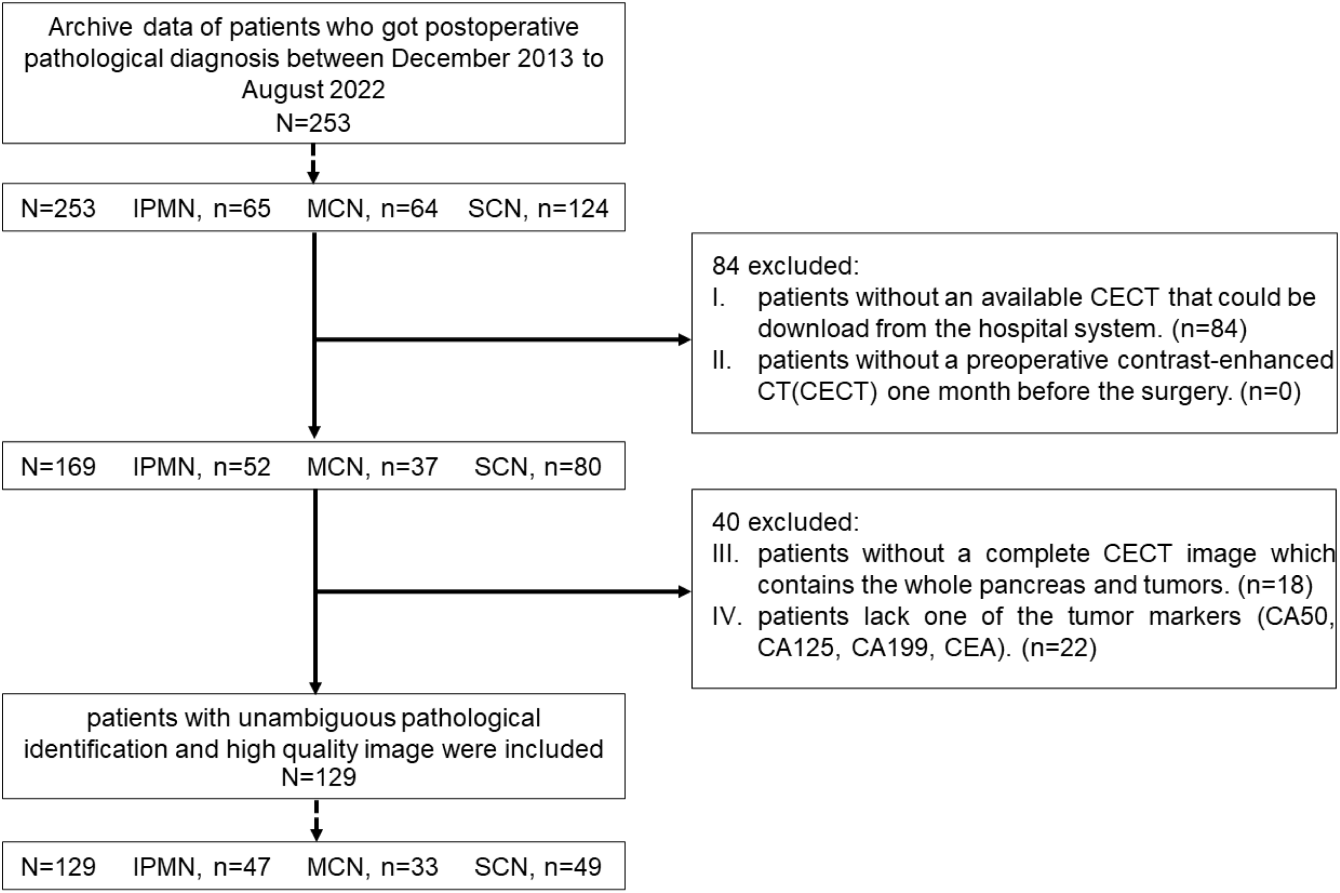
Patients’ enrolment process for this research.

This retrospective study was approved by Human Research Ethics Committee of our hospital. From December 2013 to August 2022, a total of 253 patients, including 65 IPMN cases, 124 SCN cases, and 64 MCN cases, were pathologically confirmed as patients with PCN. All the patients were screened by the following exclusion criteria (shown in **Figure 2**.): (i) patients without an available contrast-enhanced CT (CECT) that could be downloaded from the hospital system; (ii) patients without a preoperative CECT 1 month before the surgery; (iii) patients without a complete CECT image that contains the whole pancreas and tumors; and (iv) patients lack one of the following tumor markers [CA50, CA125, CA199, and carcinoembryonic antigen (CEA)]. Finally, 129 patients, including 47 IPMN cases, 49 SCN cases, and 33 MCN cases, were included in this research.

### CT protocols

Patients were told to take quiet respiration to minimize the artifact disturbance. Then, CT examination was performed on a 64-detector spiral CT-system (Somantom Definition AS, Siemens, Erlangen, Germany), providing acquisition capability of 128 slices with the following scanning parameters: 120 kV, 280 mA, 0.33-mm slice thickness with an interval of 0.33 mm, 1.5 spiral pitch, and tube rotation cycle of 0.33 s. The four-phase CT images included (1) plain scan (2), arterial phase (20 to 25 s of delay) (3), venous phase (50 to 55 s of delay), and (4) delay phase.

### Segmentation

**Figure 3:**
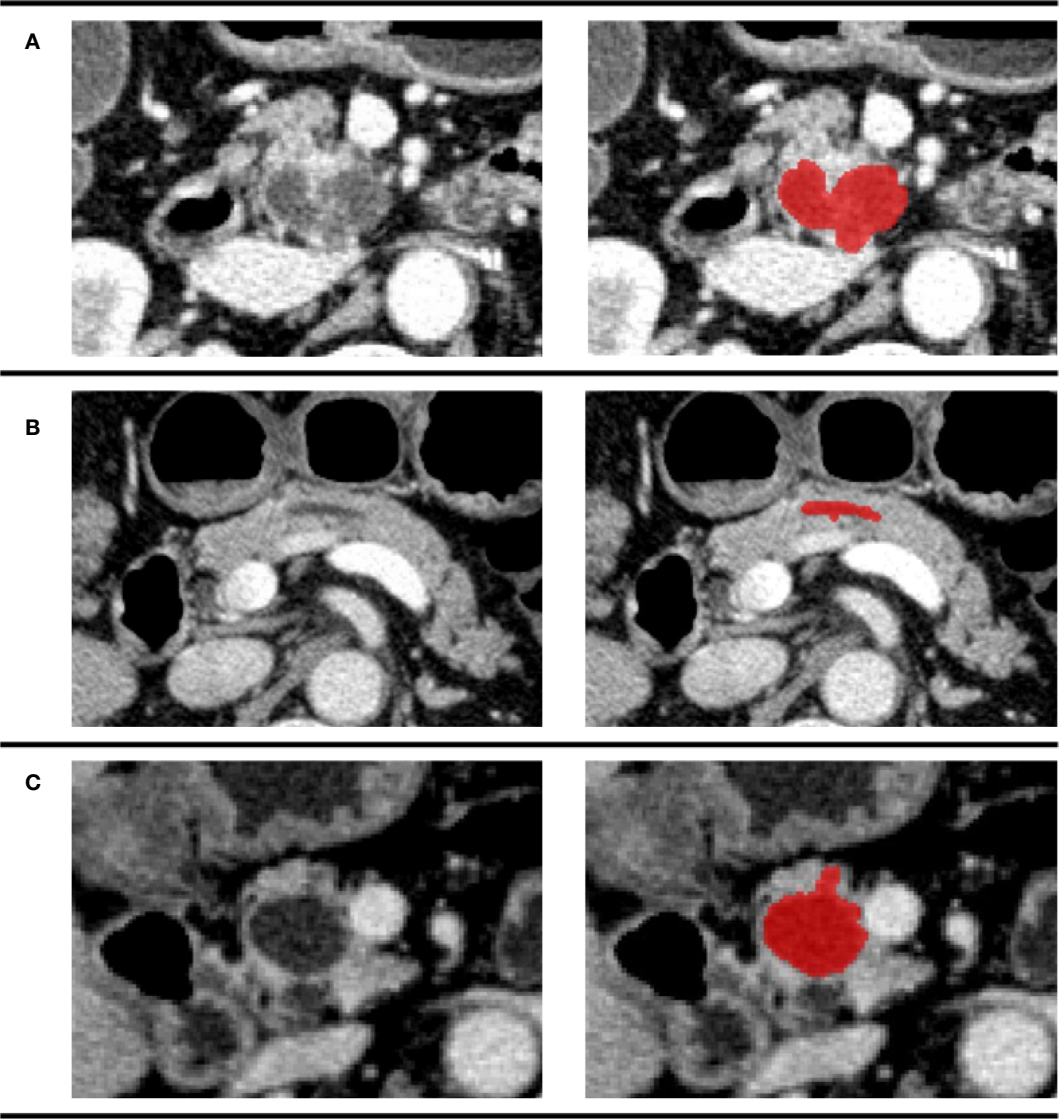
Examples of different patients in the venous phase. Tumor regions were drawn with red. **(A)** CECT image of a 74-year-old man pathologically diagnosed with IPMN; **(B)** CECT image of a 61-year-old woman pathologically diagnosed with SCN; **(C)** CECT image of a 70-year-old man pathologically diagnosed with MCN.

All the image segmentations were independently performed by two 5-year experienced pancreatic surgeons. The readers were both blinded to the pathologic result. Cases were divided equally to two readers. After all the work was done, images were exchanged between readers and checked again. Any disagreement would be sent to a director who would make the final decision. The three-dimensional (3D) Region of Interest (ROI) were completed by ITK-SNAP (version 3.8.0). According to the National Comprehensive Cancer Network (NCCN) guidelines (version 2.2019, 9 April 2019), venous phase images were recommended as the best phase for diagnosis (20). Examples of different patients in the venous phase are shown in **Figure 3**.

### Data preprocessing

First, truncate the gray value of the original data to [−100, 240] empirically, which still can fully cover the pancreas in the scans. Then, normalize it into the range [−1, 1]. Second, resample all samples to 1 × 1 × 1 mm according to the physical resolution of the original image. Third, perform the resample of CT image by bilinear interpolation from XY plane and XZ plane with python and OpenCV package.

### Radiomics feature extraction methodology

Five steps were applied to extract the radiomics feature.

**Figure 4:**
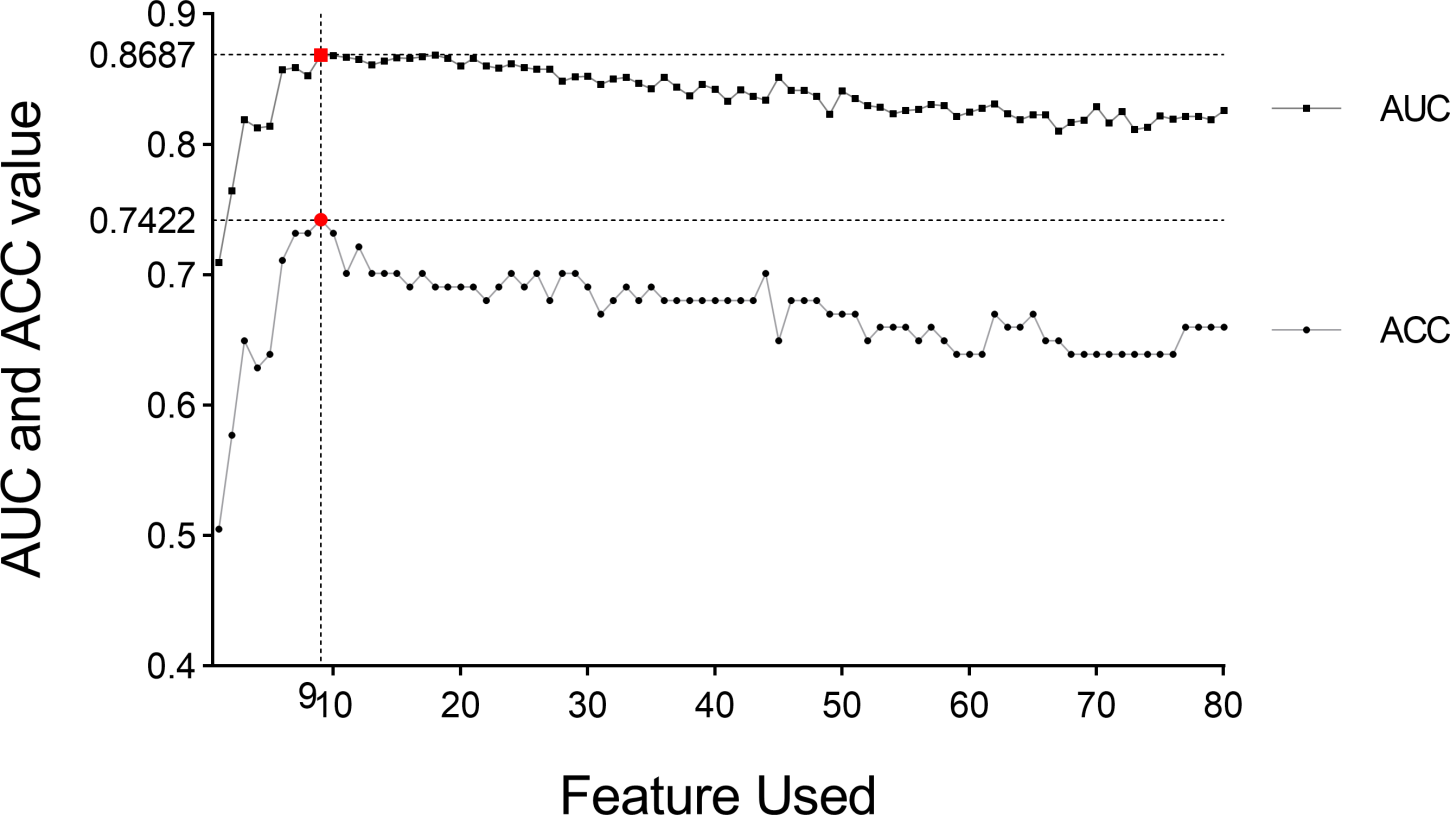
AUC value and diagnosis accuracy constructed with the number of used features. The optimal feature number was 9 with the highest ACC value of 74.22% and AUC value of 86.87% in Model 6. Moreover, the respective AUC values of IPMN, SCN, and MCN have also reached 0.9083, 0.8622, and 0.8356 (**Figure 7**). We find that the overall predictive rate was 74.23% and the predictive rates in IPMNs, SCNs, and MCNs were 74.26%, 78.37%, and 68.00%, respectively. Confusion matrix was also drawn in **Figure 7**. The sensitivity (SEN) rates of IPMN, SCN, and MCN were 74.29%, 78.38%, and 68.00%. The specificity (SPEC) rates of IPMN, SCN, and MCN were 91.94%, 81.67%, and 87.50%.

1. Randomize the included cases into the training group and the test group. In each disease, 25% of the cases were randomly selected into the test group (**Table 1**). 2. Calculate the 3D images features by the Pyradiomics package (python 3.6) (21) and statistics clinical features of the dataset. 3. Use DXScore algorithm as follows to arrange the feature: ① Calculate each feature’s DXScore. Its mathematical expression is $D\left(X\right)={\left(m_{positive}-m_{negative}\right)}^{2}/\left(d_{positive}^{2}+d_{negative}^{2}\right)$ where *m* and *d* are the mean value and standard deviation of the feature X to positive (or negative) samples (22, 23); ② Arrange features in a descending order of the score. 4. Select features and construct model. The features were assessed with SVM in the training group with five-fold cross-validation, which is an effective method for validating overfitting (24, 25). Briefly, select the first N features, in turn, to assess the classification performance by five-fold cross-validation using SVM to obtain the relation of the number of features and the accuracy of classification (as shown in **Figure 4**). First, N features were selected under the maximum accuracy. Repeat the fourth step above 10 times to verify the stability of our method. 5. The test group was applied to verify the accuracy and robustness of the model again.

**Table 1:** Characteristics of patients with IPMNs, SCNs, and MCNs.

	IPMNs	SCNs	MCNs	*P*-value
**Number of patients** **(training group, test group)**	47 (35, 12)	49 (37, 12)	33 (25, 8)	
**Age (mean, range)**	64.15 (36–83)^#*^	57.90 (27–77)^&^	52.54 (25–79)	9.3 × 10^−5^
**Gender**	^#*^			4.0 × 10^−6^
Male	30 (63.83%)	14 (28.57%)	4 (12.12%)	
Female	17 (36.17%)	35 (71.43%)	29 (87.88%)	
**Tumor location**	^#*^			1.3 × 10^−5^
Head	28 (59.57%)	11 (22.45%)	4 (12.12%)	
Neck	11 (23.40%)	11 (22.45%)	5 (15.15%)	
Body	5 (10.64%)	9 (18.37%)	9 (27.27%)	
Tail	3 (6.38%)	18 (36.73%)	15 (45.45%)	
**Tumor marker** **(mean, range)**				
CA50	25.05 (0.50, 267.10)	15.75 (0.83, 74.67)	14.34 (2.38, 87.79)	0.192
CA125	27.62 (5.02, 555.30)	5.75^&^ (0.35, 25.60)	19.29 (1.30, 95.01)	0.087
CA19-9	46.20 (2.00, 799.20)	8.88^&^ (0.35, 80.90)	23.99 (2.93, 117.00)	0.044
CEA	3.16^#^ (0.91, 25.21)	13.79^&^ (0.31, 180.0)	1.69 (0.41, 4.30)	0.001

# indicates significant difference between IPMNs and SCNs; * indicates significant difference between IPMNs and MCNs; & indicates significant difference between SCNs and MCNs.

Age^#^, P = 0.012; Age*, P = 2.0 × 10^−5^; Age^&^, P = 0.033; Gender^#^, P = 5.3 × 10^−4^; Gender*, P = 4.0 × 10^−6^; Tumor location^#^, P = 2.5 × 10^−4^; Tumor location*, P = 4.0 × 10^−6^; CA125^&^, P = 5.1 × 10^−4^; CA19-9^&^, P = 0.016; CEA^#^, P = 0.022; CEA^&^, P = 0.007.

### Statistical analysis

Statistical analysis and graph drawing were performed using SPSS version 26.0 (IBM SPSS Inc., Chicago, IL, USA) and GraphPad prism version 9.4.1 (GraphPad Software., San Diego, CA, USA). *P-*value below 0.05 is considered statistically significant.

## Result

### Patient information

A total of 129 patients, including 47 IPMNs, 49 SCNs, and 33 MCNs, were analyzed in this study. The difference between age (*P* = 9.3 × 10^−5^), gender (*P* = 4.0 × 10^−6^), tumor location (*P* = 1.3 × 10^−5^), CA125 (*P_SCNs/MCNs_* = 5.1 × 10^−4^), CA19-9 (*P* = 0.044), and CEA (*P* = 0.001) was statistically significant among the three categories. More detailed information about the characteristics of the patients is shown in **Table 1**.

### Selection of radiomics and clinical features

A total of 950 features, including six significant features (age, gender, tumor location, CA125, CA19-9, and CEA) in patients’ clinical information, and 944 features from Pyradiomics documentation that could be categorized as seven classes (**Figure S1**) were extracted in the training group in Section 2.6. The features were ordered by a DXScore value in a descending order. More detailed information about ordered features is shown in **Table S1**.

### Construction and validation of radiomics model

**Figure 5:**
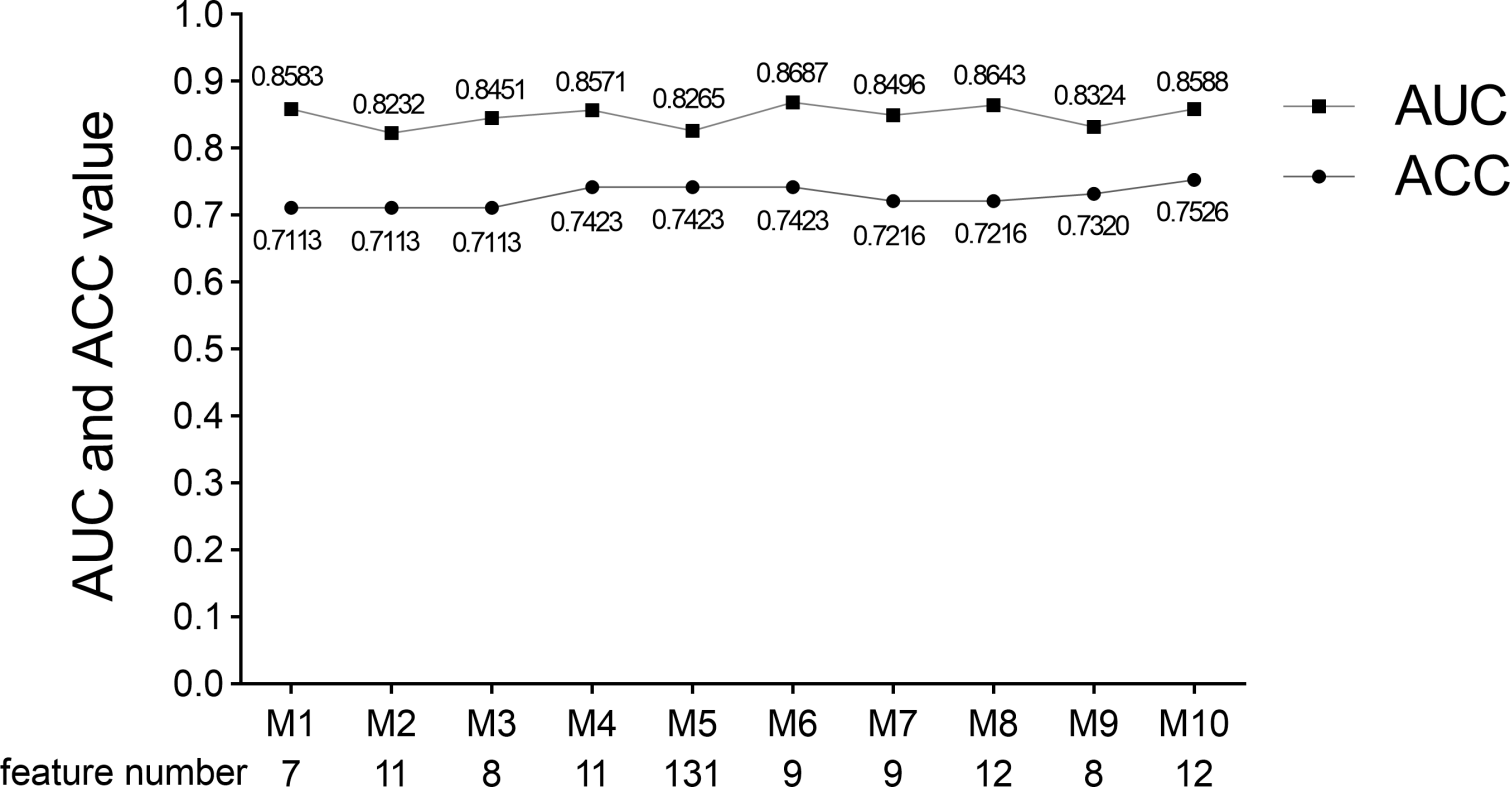
AUC value, ACC value, and the used feature number of 10 times five-fold cross-validation models. Model 6 was selected because of its suitable feature number and slightly higher AUC and ACC values. M indicates Model.

**Figure 6:**
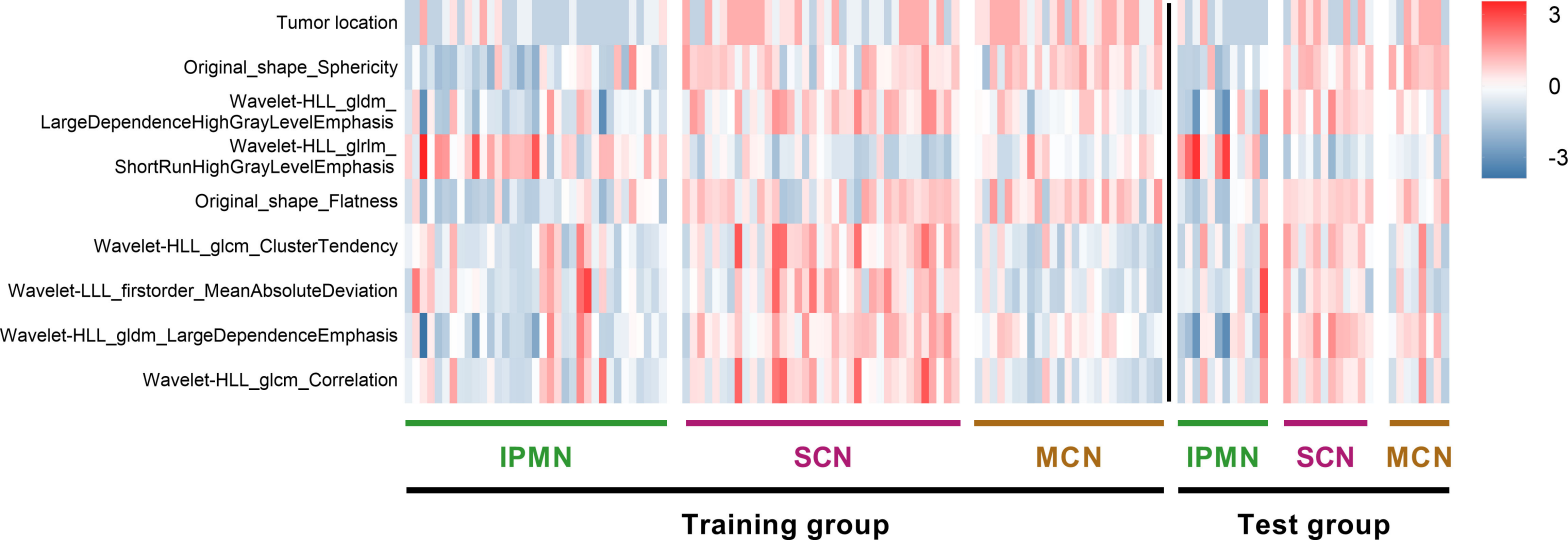
Heat map of radiomics features and cases in the three categories. The features on y-axis from top to bottom are arranged in a descending order according to the DXScore value. Right color bar represents color coding of normalized value of each radiomics feature.

We performed five-fold cross-validation 10 times to construct models. The standard deviation of area under the receiver operating characteristic curve (AUC) value (0.0152) and accuracy (ACC) value (0.0146) proved the models’ robustness and reliability. Balanced with the number of the used features, diagnostic accuracy, and AUC value in each model, we finally chose Model 6 for the following analysis (**Figure 5**). The relationship between the number of the used features and the diagnostic accuracy in Model 6 is shown in **Figure 4**. All the selected features’ information is shown in **Figure 6**.

**Figure 7:**
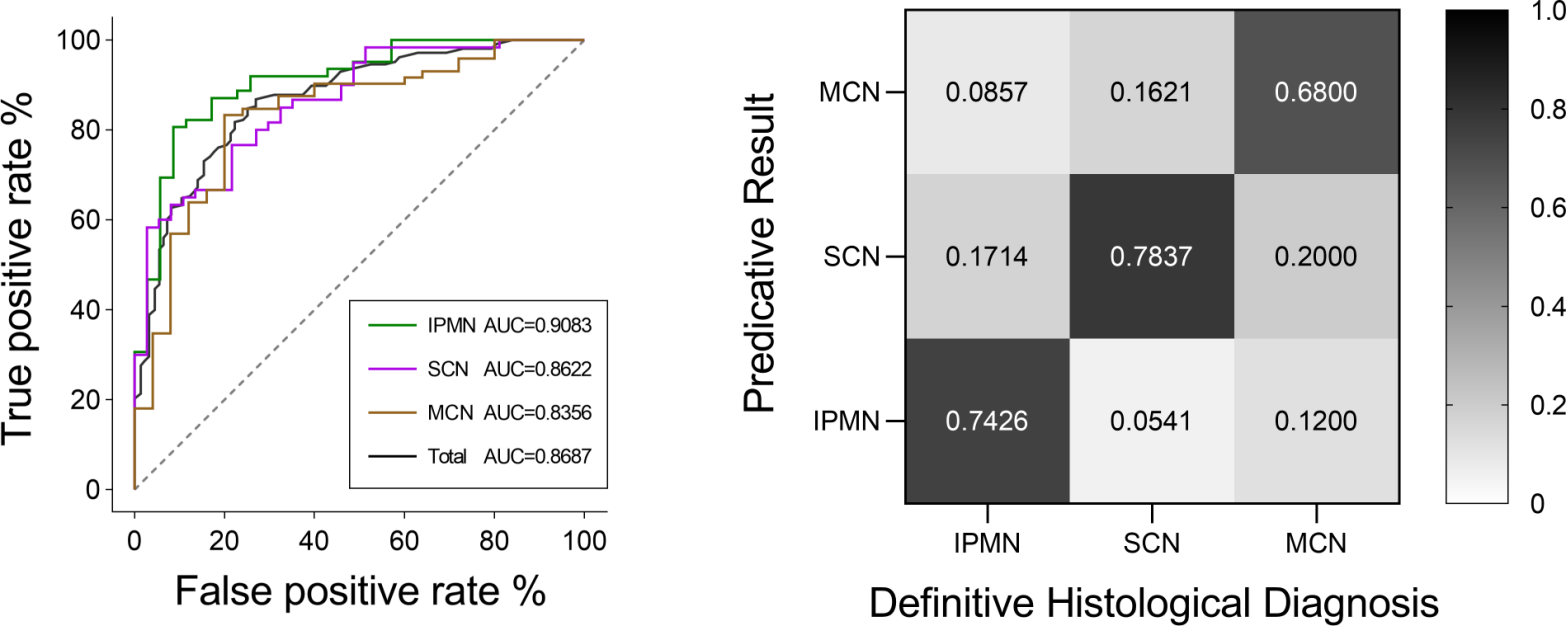
ROC curves of IPMN, SCN, and MCN and the total cases for the Model 6. The final AUC values of IPMN, SCN, and MCN were 0.9083, 0.8622, and 0.8356, respectively. In addition, the mean AUC value of five-fold cross-validation was 0.8687. ROC, receiver operating characteristic; AUC, area under the ROC curve. Relative confusion matrix of the three categories. Right color bar represents color coding of predicative rates from 0 to 1.

And the respective AUC values of IPMN, SCN, and MCN have also reached 0.9083, 0.8622 and 0.8356 (**Figure 7**). We are were delighted to find that the overall predictive rate was 74.23% and predictive rate in IPMNs, SCNs and MCNs were 74.26%, 78.37% and 68.00%, respectively. Confusion matrix was also drawn in **Figure 7**. The sensitivity of IPMN, SCN, and MCN were 74.29%, 78.38% and 68.00%. The specificity of IPMN, SCN, and MCN were 91.94%,81.67% and 87.50%.

**Figure 8:**
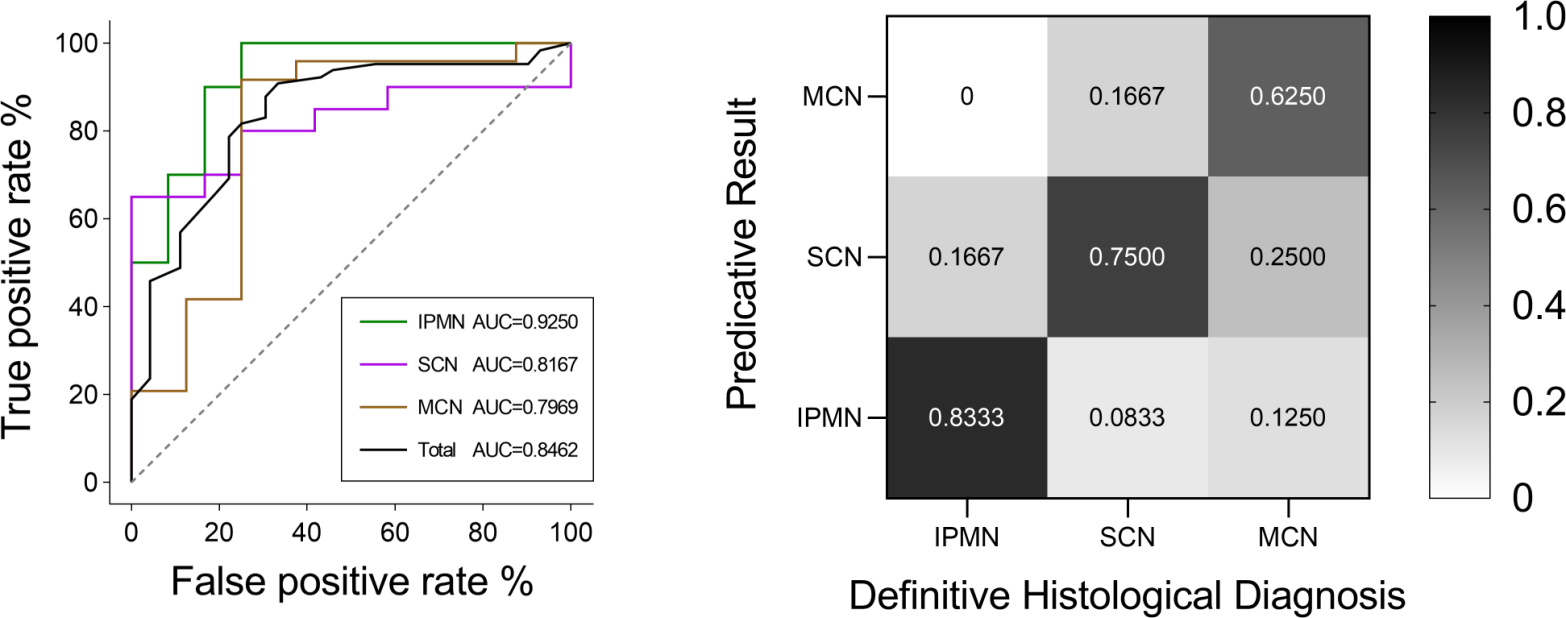
ROC curves of IPMN, SCN, and MCN and the total cases in the test group. The final AUC values of IPMN, SCN, and MCN were 0.9250, 0.8167, and 0.7969, respectively. In addition, the mean AUC value of test group was 0.8462. ROC, receiver operating characteristic; AUC, area under the ROC curve. Relative confusion matrix of the three categories. Right color bar represents color coding of predicative rates from 0 to 1.

The classification model was conducted on the test group to test its robustness and generalization. The ROC curves and detailed information regarding diagnostic discrepancies between model’s predictive result and definitive histological diagnosis in the test group are shown in **Figure 8**. In brief, the overall AUC value and predictive rate were 0.8462 and 73.61%, respectively, which illustrated the reliability of our model. The SEN rates of IPMN, SCN, and MCN in the test group were 83.33%, 75.00%, and 62.50%. The SPEC rates of IPMN, SCN, and MCN in the test group were 95.00%, 80.00%, and 87.50%.

Least absolute shrinkage selection operator (LASSO) regression is widely used because of its well performance (26–29). We compared DXScore algorithm with LASSO regression in the test group. ACC, AUC, SEN, and SPEC rates were all higher in DXScore algorithm than that in LASSO regression, especially 13.93% higher in ACC (**Table 2**).

**Table 2:** SVM classification performance of selected feature subsets with different methods in the test group. .

Method of Feature Selection	Number of Selected Features	ACC	AUC	SEN	SPEC
DXScore	9	0.7361	0.8462	0.6944	0.8522
LASSO	16	0.5938	0.7590	0.5694	0.7944

ACC, accuracy; AUC, area under the ROC curve; SEN, sensitivity; SPEC, specificity; LASSO, least absolute shrinkage selection operator.

To conduct a deeper and more detailed research, we also analyzed the relationship between selected features and each patient quantitatively. The distribution of the nine selected radiomics features in 129 cases, categorized as IPMNs, SCNs, and MCNs, is shown in **Figure 6**. For a better visualization, we normalized each value of radiomics features by mean and standard deviation. As is shown in the heat map, the higher the DXScore value of the features was, the deeper the color was used, and distinct patterns among three categories can be observed.

## Discussion

Patients with PCN have distinct treatment principles. Patients with SCNs demonstrate benign preponderance and a good prognosis (30, 31). Patients are recommended with regular monitoring and follow-up (32). MCNs, main pancreatic duct IPMNs, and mixed IPMNs show a higher risk of malignant transformation (33, 34). Surgery is recommended to those patients before the neoplasms progress to cancer (35). Although it is the most common type of IPMNs, BD-IPMN has a relatively low malignant tendency and it does not often invade the main pancreatic duct. Because of subtle difference in imaging characteristics, it is currently extremely difficult to perform differential diagnosis through traditional CT and MRI scans. According to the previous studies, the overall preoperative diagnostic accuracy rates of PCNs by clinicians were 37.3% and 61.0%, with SCN diagnostic accuracy rates of 30.4% and 24.2%, which is far from satisfactory (26, 36). Thus, we retrospectively analyzed 129 patients and constructed a DXScore algorithm–based model with an overall accuracy rate of 74.23%.

In our results, we have screened out a few important features. Among those radiomics features, “Original shape Sphericity” was the most important one. Sphericity is a measure of the roundness of the shape of the tumor region relative to a sphere (37). In general, non-SCNs demonstrate a regular oval shape and usually hold a smooth contour, whereas SCNs tended to have a multicystic or lobulated shape. Sahani et al. and Kim et al. also discovered the importance of surface contours in diagnosing PCN (38, 39). Because the following seven radiomics features, included in model (**Figure 6**), were obtained through wavelet and principal component analysis that cannot be directly reflected in the original images, there was no intuitive clinical implication. As for the clinical features, tumor location was selected by DXScore algorithm, ranking first among the nine features. Indeed, the majority of IPMN is located in the head of the pancreas in traditional analysis, whereas SCN and MCN are often localized in the body or tail of the pancreas (40–43). Tumor location may have a more intuitive clinical implication than other clinical features in PCNs diagnosis. Whereas, five significant ones (age, gender, CA125, CA19-9, and CEA) were not included in the machine learning algorithm. Wei et al. found that gender was an important SCN diagnostic factor (26). Moreover, there was a controversial in the relationship between age and PCNs. Kim et al., Goh et al., and Wei et al. found that age was an insignificant differential diagnostic factor. Whereas, several other studies have considered age as an important factor (38, 44, 45). However, those were finally not selected by DXScore in our study. Therefore, this is a typical example that artificial intelligence is able to surpass people’s inherent logic and achieve a better classification result.

For a more precise model and automatic process, there would be two steps: first is the automatic identification of the pancreas and second is the construction of models with higher accuracy. Manual segmentation takes time and effort. Although the accuracy of automatic pancreas segmentation has been up to 85%, there is a long way before automatic pancreas segmentation can be applied in clinical practices (46–48). For our sake, we will further explore in the field of automatic pancreas segmentation to accelerate the process of clinical application of automatic pancreatic disease identification. As for classification algorithm, several methods have been widely applied in various research studies: Wilcoxon rank-sum test (WRST), relief, logistic regression, X^2^-test, and LASSO. In our study, we applied a novel DXScore algorithm. Compared with an AUC value of 0.7590 for LASSO in our research, DXScore algorithm achieved an AUC value of 0.8462.

In the field of PCN differential diagnosis, most literatures constructed a binary classification model (26, 28, 49). Only Dmitriev et al. constructed a four-class classification model for the diagnosis of IPMNs, MCNs, SCNs, and solid pseudopapillary neoplasms with convolutional neural network and random forest classifier. This model reached a diagnostic rate of 95.9%, 64.3%, 51.7%, and 100%, respectively (50). In our ternary classification model, the diagnostic rates in the five-fold cross-validation algorithm of IPMNs, MCNs, and SCNs have reached 74.26%, 78.37%, and 68.00%, respectively, and the diagnostic rates of IPMNs, MCNs, and SCNs in the test group have reached 83.33%, 75.00%, and 62.50%, respectively. Our model has shown a feasible performance in the differential diagnosis between SCNs and MCNs, which is the most difficult one according to the clinical experience.

Our article also has some limitations. Because of the ternary classification model, a relatively small number of patients were included in each category. Because most patients with pancreatic cysts have no clinical symptoms and not all the patients require surgical intervention, it is difficult for a single center to obtain a large number of pathologically identified cases (51, 52). In the next step, we will continue to recruit patients, improve the PCNs database capacity, and further test the extensibility of our model with multicenter data.

In conclusion, this research preliminarily verified the application value of radiomics in the differential diagnosis of pancreatic cystic tumors. With more intensive future research and the construction of more reliable prediction models, artificial intelligence technology will greatly help clinicians in the diagnosis and treatment of diseases. In the future, a large number of studies are still needed to conduct prospective studies to further confirm the diagnostic accuracy and application value of imaging omics in clinical practices.

## Data availability statement

The raw data supporting the conclusions of this article will be made available by the authors, without undue reservation.

## Ethics statement

The protocol and all amendments were approved by the Institutional Review Board of Renji Hospital, School of Medicine, Shanghai Jiao Tong University (Shanghai, China).

## Author contributions

ZD: Conceptualization;Methodology;Formal analysis;Writing - Original Draft;Visualization. XC: Methodology;Software;Visualization. ZC: Formal analysis;Data Curation. YL: Formal analysis;Data Curation. MH: Data Curation TC: Data Curation. ZZ: Conceptualization;Validation;Writing - Review and Editing;Supervision. XQ: Validation;Writing - Review and Editing;Supervision. WC: Validation;Writing - Review and Editing;Supervision;Funding acquisition. All authors contributed to the article and approved the submitted version.
