# Supplementary material for: Differential diagnosis of pancreatic cystic neoplasms through a radiomics-assisted system
Source: Front Oncol. 2022 Dec 16;12:941744. doi: 10.3389/fonc.2022.941744 (PMC9802410; doi:10.3389/fonc.2022.941744)
Supplement: Supplementary file 1 [file Table_1.docx]

| Rank | Feature name |
| --- | --- |
| 1 | location |
| 2 | original_shape_Sphericity |
| 3 | wavelet-HLL_gldm_LargeDependenceHighGrayLevelEmphasis |
| 4 | wavelet-HLL_glrlm_ShortRunHighGrayLevelEmphasis |
| 5 | original_shape_Flatness |
| 6 | wavelet-HLL_glcm_ClusterTendency |
| 7 | wavelet-LLL_firstorder_MeanAbsoluteDeviation |
| 8 | wavelet-HLL_gldm_LargeDependenceEmphasis |
| 9 | wavelet-HLL_glcm_Correlation |
| 10 | wavelet-HLH_gldm_DependenceEntropy |
| 11 | wavelet-HLL_glcm_ClusterProminence |
| 12 | wavelet-LLH_gldm_LargeDependenceEmphasis |
| 13 | wavelet-HHL_gldm_DependenceEntropy |
| 14 | original_firstorder_MeanAbsoluteDeviation |
| 15 | wavelet-LLH_glrlm_LongRunEmphasis |
| 16 | wavelet-HLH_gldm_DependenceNonUniformityNormalized |
| 17 | wavelet-LLL_firstorder_Variance |
| 18 | wavelet-HLH_gldm_DependenceVariance |
| 19 | wavelet-LLL_firstorder_90Percentile |
| 20 | wavelet-LLH_glrlm_RunLengthNonUniformityNormalized |
| 21 | wavelet-HLL_glcm_Contrast |
| 22 | wavelet-HLL_glcm_DifferenceAverage |
| 23 | wavelet-HLL_glcm_InverseVariance |
| 24 | wavelet-HLL_glcm_Idn |
| 25 | wavelet-HLL_glcm_Idm |
| 26 | wavelet-HLL_glcm_Id |
| 27 | wavelet-HLL_glcm_Idmn |
| 28 | wavelet-HLL_ngtdm_Complexity |
| 29 | wavelet-LLH_glrlm_LongRunHighGrayLevelEmphasis |
| 30 | wavelet-HLL_glrlm_RunLengthNonUniformityNormalized |
| 31 | wavelet-HHL_gldm_DependenceNonUniformityNormalized |
| 32 | wavelet-HHH_gldm_DependenceEntropy |
| 33 | wavelet-LLH_glrlm_RunPercentage |
| 34 | original_firstorder_90Percentile |
| 35 | wavelet-LLH_glrlm_ShortRunEmphasis |
| 36 | wavelet-HLL_glrlm_ShortRunEmphasis |
| 37 | wavelet-HLL_ngtdm_Contrast |
| 38 | wavelet-LLL_firstorder_RobustMeanAbsoluteDeviation |
| 39 | wavelet-LHH_gldm_DependenceEntropy |
| 40 | wavelet-HHL_gldm_DependenceVariance |
| 41 | wavelet-LLH_gldm_DependenceNonUniformityNormalized |
| 42 | wavelet-HLL_glrlm_RunPercentage |
| 43 | wavelet-LHH_gldm_DependenceNonUniformityNormalized |
| 44 | wavelet-LLH_gldm_DependenceEntropy |
| 45 | gradient_firstorder_RobustMeanAbsoluteDeviation |
| 46 | wavelet-LLH_glrlm_RunEntropy |
| 47 | original_firstorder_Variance |
| 48 | wavelet-LHL_glcm_ClusterTendency |
| 49 | wavelet-LHL_glcm_ClusterProminence |
| 50 | gradient_firstorder_InterquartileRange |
| 51 | wavelet-HLL_gldm_DependenceNonUniformityNormalized |
| 52 | wavelet-HHH_gldm_DependenceNonUniformityNormalized |
| 53 | wavelet-LHL_gldm_DependenceEntropy |
| 54 | wavelet-HHH_gldm_DependenceVariance |
| 55 | wavelet-LHL_glcm_Correlation |
| 56 | original_firstorder_RobustMeanAbsoluteDeviation |
| 57 | wavelet-HLL_gldm_DependenceEntropy |
| 58 | wavelet-LLH_gldm_LargeDependenceHighGrayLevelEmphasis |
| 59 | wavelet-LLH_glcm_ClusterTendency |
| 60 | wavelet-HLL_glcm_SumEntropy |
| 61 | gradient_firstorder_90Percentile |
| 62 | wavelet-LLH_glrlm_ShortRunHighGrayLevelEmphasis |
| 63 | wavelet-LLL_firstorder_InterquartileRange |
| 64 | wavelet-LHL_ngtdm_Complexity |
| 65 | wavelet-LHH_gldm_DependenceVariance |
| 66 | wavelet-LHL_gldm_DependenceNonUniformityNormalized |
| 67 | wavelet-LLH_glrlm_RunVariance |
| 68 | wavelet-LHL_glcm_Id |
| 69 | wavelet-LHL_glcm_Idm |
| 70 | wavelet-LHL_glcm_Idn |
| 71 | wavelet-LHL_glcm_DifferenceAverage |
| 72 | wavelet-LHL_glcm_Contrast |
| 73 | wavelet-LHL_glcm_InverseVariance |
| 74 | wavelet-LHL_glcm_Idmn |
| 75 | wavelet-LLH_glrlm_LongRunLowGrayLevelEmphasis |
| 76 | wavelet-LHL_ngtdm_Contrast |
| 77 | Gender |
| 78 | wavelet-LLH_ngtdm_Contrast |
| 79 | original_firstorder_InterquartileRange |
| 80 | wavelet-LLH_ngtdm_Complexity |
| 81 | original_shape_Elongation |
| 82 | original_gldm_DependenceEntropy |
| 83 | gradient_gldm_DependenceEntropy |
| 84 | wavelet-LLL_gldm_DependenceEntropy |
| 85 | original_gldm_DependenceNonUniformityNormalized |
| 86 | gradient_gldm_DependenceNonUniformityNormalized |
| 87 | wavelet-LLL_gldm_DependenceNonUniformityNormalized |
| 88 | wavelet-LLH_glcm_Correlation |
| 89 | wavelet-HLL_glrlm_RunEntropy |
| 90 | wavelet-HLL_glrlm_LongRunHighGrayLevelEmphasis |
| 91 | original_gldm_DependenceVariance |
| 92 | gradient_gldm_DependenceVariance |
| 93 | wavelet-LLL_gldm_DependenceVariance |
| 94 | wavelet-LLH_gldm_DependenceVariance |
| 95 | wavelet-LLH_glcm_Idn |
| 96 | wavelet-LLH_glcm_Id |
| 97 | wavelet-LLH_glcm_Idm |
| 98 | wavelet-LLH_glcm_Idmn |
| 99 | wavelet-LLH_glcm_InverseVariance |
| 100 | wavelet-LLH_glcm_Contrast |

**Table S1** Top 100 Features` name and rank. Feature is ranked by DXscore value.
