# Supplementary figures and images for: Differential diagnosis of pancreatic cystic neoplasms through a radiomics-assisted system
Source: Front Oncol. 2022 Dec 16;12:941744. doi: 10.3389/fonc.2022.941744 (PMC9802410; doi:10.3389/fonc.2022.941744)

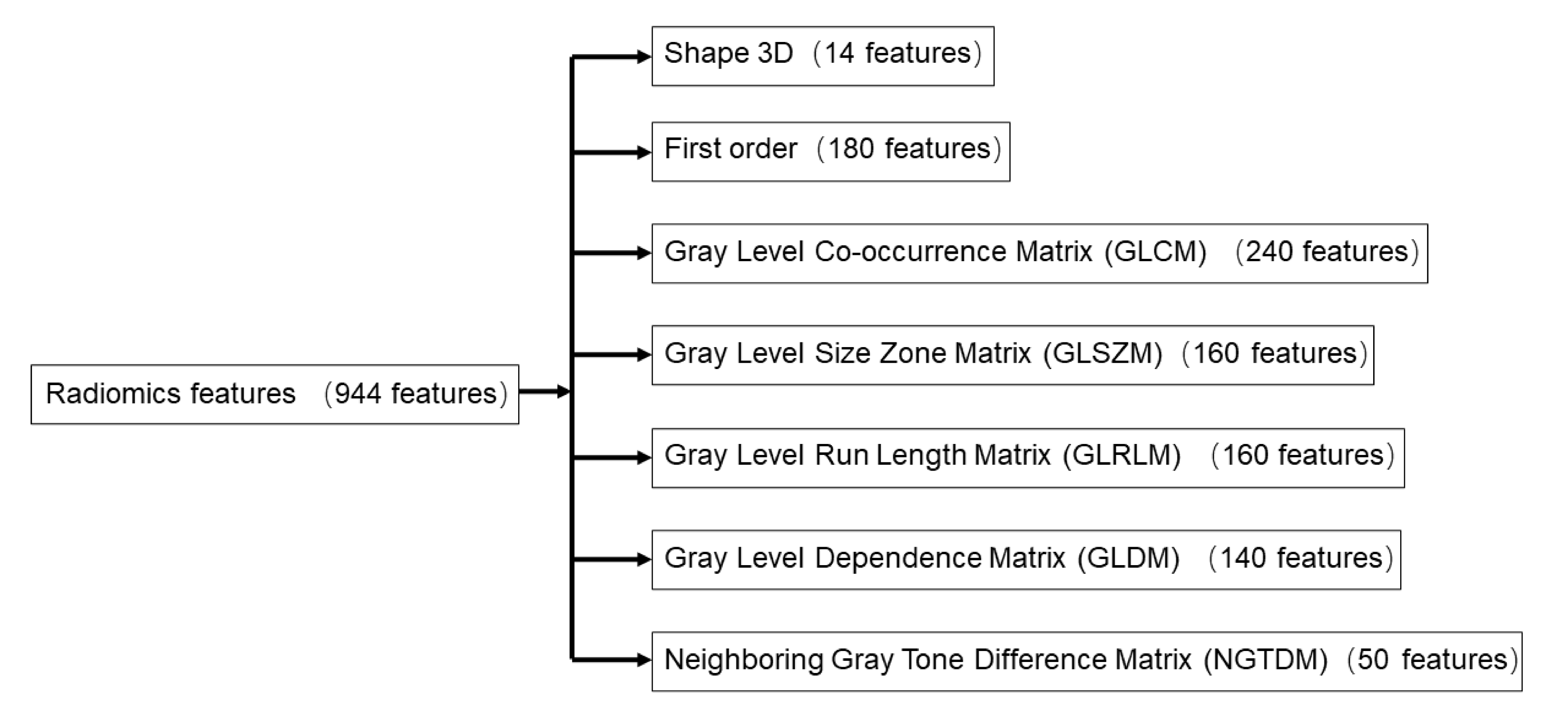

Supplement: Supplementary file 2 [file Image_1.tif]
